# Bacterial metabolites and cardiovascular risk in children with chronic kidney disease

**DOI:** 10.1186/s40348-021-00126-8

**Published:** 2021-10-22

**Authors:** Julia Schlender, Felix Behrens, Victoria McParland, Dominik Müller, Nicola Wilck, Hendrik Bartolomaeus, Johannes Holle

**Affiliations:** 1 Charité - Universitätsmedizin Berlin, corporate member of Freie Universität Berlin and Humboldt-Universität zu Berlin, Department of Pediatric Gastroenterology, Nephrology and Metabolic Diseases, 13353 Berlin, Germany; 2 Experimental and Clinical Research Center (ECRC), a cooperation of Charité - Universitätsmedizin Berlin and Max Delbruck Center for Molecular Medicine (MDC), 13125 Berlin, Germany; 3 Charité - Universitätsmedizin Berlin and Berlin Institute of Health, 10117 Berlin, Germany; 4 DZHK (German Centre for Cardiovascular Research), partner site Berlin, 13316 Berlin, Germany; 5 Institute of Physiology, Charité - Universitätsmedizin Berlin, 10117 Berlin, Germany; 6 Charité - Universitätsmedizin Berlin, corporate member of Freie Universität Berlin and Humboldt-Universität zu Berlin, Department of Nephrology and Internal Intensive Care Medicine, 10117 Berlin, Germany

**Keywords:** Chronic kidney disease, Microbiome, Uremic toxins, Children, Cardiovascular disease, Metabolism, Nutrition

## Abstract

Cardiovascular complications are the major cause of the marked morbidity and mortality associated with chronic kidney disease (CKD). The classical cardiovascular risk factors such as diabetes and hypertension undoubtedly play a role in the development of cardiovascular disease (CVD) in adult CKD patients; however, CVD is just as prominent in children with CKD who do not have these risk factors. Hence, the CKD-specific pathophysiology of CVD remains incompletely understood. In light of this, studying children with CKD presents a unique opportunity to analyze CKD-associated mechanisms of CVD more specifically and could help to unveil novel therapeutic targets.

Here, we comprehensively review the interaction of the human gut microbiome and the microbial metabolism of nutrients with host immunity and cardiovascular end-organ damage. The human gut microbiome is evolutionary conditioned and modified throughout life by endogenous factors as well as environmental factors. Chronic diseases, such as CKD, cause significant disruption to the composition and function of the gut microbiome and lead to disease-associated dysbiosis. This dysbiosis and the accompanying loss of biochemical homeostasis in the epithelial cells of the colon can be the result of poor diet (e.g., low-fiber intake), medications, and underlying disease. As a result of dysbiosis, bacteria promoting proteolytic fermentation increase and those for saccharolytic fermentation decrease and the integrity of the gut barrier is perturbed (leaky gut). These changes disrupt local metabolite homeostasis in the gut and decrease productions of the beneficial short-chain fatty acids (SCFAs). Moreover, the enhanced proteolytic fermentation generates unhealthy levels of microbially derived toxic metabolites, which further accumulate in the systemic circulation as a consequence of impaired kidney function. We describe possible mechanisms involved in the increased systemic inflammation in CKD that is associated with the combined effect of SCFA deficiency and accumulation of uremic toxins. In the future, a more comprehensive and mechanistic understanding of the gut–kidney–heart interaction, mediated largely by immune dysregulation and inflammation, might allow us to target the gut microbiome more specifically in order to attenuate CKD-associated comorbidities.

## CKD-associated cardiovascular disease in children and adolescents

The prevalence of chronic kidney disease (CKD) in children continues to increase worldwide. The reported case numbers range between 10.7 and 74.7 per million of the age-related population [1–3]. Despite ongoing efforts to improve treatment, mortality is high among patients with CKD, primarily due to cardiovascular diseases (CVD) and progression to end-stage kidney disease (ESKD) [4, 5]. Thus, there is an urgent need to identify extra-renal comorbidities at an early stage and to assess patients’ risk factors in order to effectively modify therapeutical interventions and therefore reduce mortality in children with CKD [6].

The lifespan of pediatric patients with ESKD is 30–40 years below that of healthy children [7]. While CVD is absent in healthy children, cardiovascular events account for 40–45% of deaths in children with ESKD [7–9]. The relevance of CVD in CKD has been highlighted in a statement by the American Heart Association, which classifies CKD patients in the highest cardiovascular risk stratification alongside individuals with homozygous familial hypercholesterolemia, diabetes mellitus type 1, heart transplants, or coronary aneurysms due to Kawasaki disease [4, 8, 10].

Adult CKD patients also have a drastic increase in cardiovascular morbidity [5]. In adults, the CVD is mainly driven by the primary courses of CKD: diabetes and arterial hypertension. However, these comorbidities and underlying diseases are usually absent in children with CKD. Thus, in the absence of confounding classical risk factors, cardiovascular mortality is expected to be induced largely by CKD-specific mechanisms.

This hypothesis is underlined by the observation that unlike in the aging population, pediatric patients with CKD rarely show symptomatic atherosclerosis classically associated with a consecutive calcification of the tunica intima of arteries [11]. By contrast, current data suggest the early occurrence of endothelial dysfunction in ESKD with diffuse nonocclusive medial calcification, known as Mönckeberg’s arteriosclerosis [12–14]. Medial calcification leads to arterial wall stiffening and thus reduces the compliance and elasticity of arteries resulting in increased systolic pressure and cardiac workload. Subsequently, the development of endothelial dysfunction and vascular calcification results in left ventricular hypertrophy and myocardial fibrosis, which can lead to sudden cardiac death by arrhythmia or cardiac failure [9, 15]. Recently, studies have focused on translating these morphologic findings into clinical surrogate parameters indicating the progression of CVD. In fact, a prospective observational study in more than 700 European children identified key criteria for the development of CVD such as carotid intima-media thickness (cIMT), pulse wave velocity (PWV), left ventricular mass (LVM), and vascular calcification [16–18]. Highly significant is the fact that vascular remodeling, myocardial adaptation, and arterial stiffening are clinically detectable in many children at early times when symptoms of CVD may still be absent [19].

The hypothesis of arteriosclerosis being an inflammatory disease was first postulated by Ross et al. in 1999 [20]. More recently, approaches using anti-inflammatory therapy directed against prominent cytokines, namely canakinumab against interleukin 1 beta, in the secondary prevention of arteriosclerosis have further highlighted the pivotal role of inflammation in the pathophysiology of cardiovascular complications [21]. In fact, plasma concentrations of pro-inflammatory cytokines are consistently elevated in CKD and oxidative stress is linked to inflammation by activating the nuclear factor “kappa-light-chain-enhancer” of activated B-cells (NF-κB) and inducing pro-inflammatory cytokines [22].

As it became clear that chronic inflammation constitutes one of the main risk factors of CVD [20], increasing attention has been paid to the intestinal microbiome, its metabolism, and its interaction with host inflammatory status. Although data is limited on the inflammatory status of pediatric patients with CKD, results from studies with adult patients and animal experiments highlight the interaction of the gut microbiome, kidneys, and immune system as a crucial contributor to CVD pathology [23–31].

In this review, we provide an overview of the mechanisms involved in CKD-associated alterations of the gut microbiome, the microbially produced metabolites, and their relation to the development of CVD. Particularly, we focus on bacterial and host metabolism of amino acids, such as tryptophan (TRP) and tyrosin (TYR), as well as the influence of short-chain fatty acids (SCFAs). While of limited epidemiological importance, research in pediatric CKD patients enables the investigation of CVD mechanisms specific to kidney disease independent of other risk factors and therefore can extend our pathophysiological understanding of CVD in CKD. Furthermore, we discuss novel therapeutic approaches based on the understanding of the microbiome and its metabolites that could help lower the enormous burden of cardiovascular morbidity and mortality in patients suffering from CKD.

## The intestinal microbiome and microbiome–host interactions in health and CKD

The microbiome is defined not only by a community of microorganisms living in a defined environment (microbiota), but also by the whole spectrum of molecules produced by the microorganisms (including nucleic acids and metabolites) and the surrounding environment [32].

In 2007, the Human Microbiome Project was launched to improve the understanding of the enormous diversity of microbial flora. Most strikingly, it refuted the assumption that humans share a large core of microbial taxa, sprinkled with a few lineages that make each individual unique. Quite the contrary, interindividual differences are substantially greater than previously expected [33–37]. This observation was consolidated by the European Union project METAgenomics of the Human Intestinal Tract (MetaHIT). This study showed that a microbial species found with up to 10% abundance in one individual can be as rare as one cell in 1000 in another participant within the cohort. In summary, these findings indicate an extraordinarily complex and dynamic consortium of bacteria residing in the human gut which consistently responds to internal and external stimuli [38–40]. Despite substantial differences in the abundance of microbial species in the human gut microbiota, a larger overlap of genes encoding for metabolic functions exists [41]. Taken together, healthy microbiomes may differ significantly in species abundance, but the metabolic potential of the gut microbiome from different individuals share common overlapping features.

## Bacteria–host interaction: how the gut microbiome is shaped throughout life

During the human lifetime, the intestinal flora undergoes dynamic changes with the most substantial alterations occurring during childhood [42, 43]. Recently, it has been shown that meconium before birth is indeed sterile; however, contrary to a widespread assumption, newborns are not sterile at birth. In fact, the vaginal flora evolves throughout pregnancy to provide the newborn with beneficial microbes, such as Lactobacillus and Prevotella which are transmitted to the child during birth [44–46]. These maternally provided microbial communities occupy niches and protect newborns from the colonization of pathogens. Interestingly, while children born through vaginal delivery share a significant proportion of 16S rRNA sequences with their biological mother for up to 2 years, the gut residing in the gut of children born via cesarean section (C-section) is more similar to the hospital environment and the mother’s skin. This may contribute to the 64–82% higher risk to sustain skin infections with methicillin-resistant Staphylococcus aureus in the case of neonates delivered via C-section compared to vaginal birth. Although not proven yet because of the high risk of confounding factors, the altered composition of gut microbiota that results from C-section births may be associated with an increased risk of obesity, atopic diseases like asthma, and Crohn’s disease in later life [47–52].

Despite the mode of birth, breastfeeding is among the most influential perinatal factors shaping the intestinal microbiome composition, partly by providing both a source of beneficial commensals, including *Staphylococci*, *Streptococci*, lactic acid bacteria, and *Bifidobacteria* [53], and human milk oligosaccharides as an energy source for beneficial gut bacteria [54]. In the neonatal period, the composition of the gut microbiome is very dynamic, starting at low levels of distinguishable taxa, dominated by Proteobacteria and Actinobacteria and successively changing toward a more diverse population with Firmicutes and Bacteroidetes emerging and dominating the local environment in older individuals [36, 55]. These life changes are reflected in a noticeable difference in the Firmicutes/Bacteroidetes ratio between infants and adults (ratio 0.4 and 10.9, respectively). Interestingly, ratios in the elderly tend toward values more similar to those in infants [56–58].

However, the relationship between the gut microbiome and health and disease is complex. Our knowledge of variation in the intestinal microbiome composition across the human lifetime remains, at best, superficial.

Whereas large cohort studies provided in-depth data to generate a microbiome profile in the adult population, much less is known about the microbiome of children and adolescents [59]. A recent study involving 16S rRNA sequencing of microbiome fecal samples from 2111 children in the age range of 9 to 12 years revealed a significantly lower Shannon diversity than the adult control group suggesting that the immature microbiome fundamentally differs from that of adults. Metagenome analysis provided additional information showing that these compositional differences also reflect in functional metabolic disparities including the overrepresentation of vitamin B biosynthesis pathways (riboflavin: B2, pyridoxine: B6, and folate: B9) and a predominance of catabolic amino acid metabolism (Valin, Leucin, and Isoleucin) in children [59, 60]. Once established, the human gut microbiome remains relatively stable over time. However, a plethora of factors can impact the gut microbiome: medication, which is not limited to antibiotics [50, 61–63], but also includes proton pump inhibitors [64, 65] and laxatives; diet (fiber, artificial sweeteners, and sodium chloride intake) [66–68]; chronic autoimmune, inflammatory, metabolic, and neurodegenerative diseases [69–72]; genetics [73]; stress [74]; exercise [75]; surgeries [76]; geography [77]; and aging itself [78, 79]. In summary, it is commonly accepted that many influences shape the gut microbiome over time.

## The benefits of symbiosis in the gut microbiome of healthy individuals

The host–microorganism relationship can generally be defined as symbiotic as the respective partners not only coexist without detriment but benefit from each other. The resident bacteria metabolize dietary components, which are otherwise inaccessible for humans, such as fiber. The microbiota is not only beneficial in the digestion of complex polysaccharides but also a key player in amino acid homeostasis (lysine and threonine), absorption of vitamins (vitamin K and B groups), metabolism of bile acids, and integrity of the intestine barrier and protecting against pathogens [31, 80–84]. In addition, the gut microbiome is crucial for host immune system maturation. This has been clearly demonstrated in animal studies, whereby mice devoid of a gut microbiota have an immature immune system phenotype with low lymphocyte counts and diminished cytokine production, which could be reversed within 3 weeks by restoring normal flora [85, 86].

Recent technological advances enabling functional readouts provide an in-depth understanding of the physiological state of an organism via genomics, proteomics, and metabolomics approaches by detecting small molecule substrates and intermediates of metabolism. In addition, the use of germ-free animal models complements the multi-omics approaches and provides insights into fundamental mechanisms of host–microbiome interactions [87]. Despite the advances in understanding microbial taxonomic composition, we are just beginning to assemble the necessary experimental and computational tools to understand the functional metabolic capacities of the gut microbiome. The bacterial genome of the gut vastly exceeds the complexity of the human genome, with many levels of potential further diversity resulting from branching and combinations of compounds like lipids and oligosaccharides. Thus, thanks to rapid advances in technology, we are at the leading edge of understanding the interplay of gut microbiota, metabolic changes, and the immune system [39, 88].

### Saccharolytic and proteolytic fermentation processes

Were it not for bacterial fermentation by the gut microbiota humans would not be able to extract nutrients and healthy beneficial compounds from dietary fiber [89].

There are two main pathways of bacterial fermentation: saccharolytic and proteolytic fermentation. Saccharolytic fermentation takes place primarily in the proximal colon [90] and involves the extraction of energy from complex oligosaccharides (α-glucans and non-α-glucan oligosaccharides) and polysaccharides (resistant starch, inulin, pectin, and cellulose), all of which are characterized by beta-glycosidic links that cannot be processed by human enzymes.

The saccharolytic fermentation of complex carbohydrates produces SCFAs. The most abundant with 60% being acetate—consisting of two carbon molecules (C2), followed by propionate (C3) at 25%, and butyrate (C4) at 15% [91–94]. SCFAs are naturally produced in small quantities in the liver; however, the proximal colon is their primary site of production [95]. A growing body of evidence suggests numerous mechanisms whereby SCFAs facilitate effects on gut health, including nutrient supply [96], gut motility [97], barrier function of colonocytes [31, 98], and “competitive exclusion” (paraphrasing the limited access of pathogenic bacteria to the gut epithelium due to expanding commensal bacteria [99])*.* Over and above this, numerous studies suggest that SCFAs modulate an individual’s inflammation state by affecting recruitment, trans-migration, and cytokine production of immune cells [31, 100–104]. However, the functional effects of these gut-derived metabolites on endothelial and immune cells within the intestine remain elusive [104, 105].

Whereas the major proportion acetate and propionate are rapidly absorbed and utilized by colonocytes or metabolized by the liver [96, 99, 105, 106], a comparatively low concentration of butyrate is found in the systemic circulation. Commonly, this is explained by the fact that the four-carbon molecule is a major energy substrate of colonocytes, thus being primarily utilized locally [96]. The dependence of colonocytes on bacterial-derived SCFAs was elegantly demonstrated in a study by Donohoe et al. who showed an impaired metabolic state in the colon of germ-free mice as measured by ATP depletion and autophagy in gut epithelial cells, which could be reversed by butyrate supplementation [107]*.*

Proteolytic fermentation, as its name implies, is the breakdown of protein in the absence of oxygen, mainly within the distal part of the colon. It has been shown that several metabolites resulting from peptides that escape digestion in the small intestine are precursors of harmful toxins including ammonia as well as thiols, phenols, and indoles [31, 99]. Consequently, it was postulated that a saccharolytic fermentation pattern is more favorable and that an imbalance toward more proteolytic bacterial activity could indeed be a pathophysiologic contributor in various diseases such as CKD.

## CKD-associated alterations in the gut microbial composition: dysbiosis

It was proposed that the accumulation of uremic toxins profoundly changes the biochemical environment in the gut and exerts a selection pressure that may favor microbes capable of using these substrates [108–110]. Vaziri et al. were the first to describe quantitative and qualitative alterations in the composition of gut microbiota in patients with CKD [69]. Since then, significant differences in the abundance of several bacterial species have been demonstrated in patients with CKD and their adverse consequences gained increasing attention. There is a general consensus that expansion of proteolytic bacteria with urease, p-cresol-producing enzymes, and indole-forming enzymes contributes to the production of nitrogen-containing compounds and, consequently, the accumulation of uremic toxins in CKD. Relevant proteolytic bacterial families include Ruminococcacae (phylum Firmicutes), Enterobacteriaceae (phylum Proteobacteria), and Pseudomonadaceae (phylum Proteobacteria). In addition, saccharolytic bacteria with enzymes essential for SCFA production, i.e., *Roseburia* (phylum Firmicutes), *Bifidobacterium* (phylum Actinobacteria), Prevotellaceae (phylum Bacteroidetes), and Lactobacillaceae (phylum Firmicutes), are depleted in patients with ESKD in comparison to healthy controls [26, 69, 108, 111–114]. In conclusion, these findings indicate a shift toward a proteolytic fermentation pattern that further contributes to the accumulation of uremic toxins fueling inflammation and eventually resulting in CVD and disease progression.

While adaptions of the intestinal flora to CKD-characteristic micro-environmental changes have been extensively reviewed in adult populations, to the best of our knowledge, only two small cohort studies exist that focus on alterations of the intestinal flora in children with CKD.

In 2015, the Midwest Pediatric Nephrology Consortium study highlighted a significant decrease in bacterial diversity in peritoneal dialysis (PD) patients when compared to healthy controls. Additionally, the composition of bacterial communities showed marked separations between pediatric CKD patients and healthy controls. Whereas at a family level the relative abundance of Proteobacteria and Enterobacteriaceae was increased, Bifidobacteria decreased in PD and transplant patients. Both findings are generally in line with results in adult microbiome studies in CKD [115, 116]. Pediatric PD patients exhibit an increase in the relative abundance of proteolytic Proteobacteria and, at a family level, Enterobacteriaceae. Furthermore, evidence for depleted amounts of beneficial SCFA-producing bacteria, including Actinobacteria and Bifidobacteriacae, has been presented at least at the RNA level [115, 116].

### Factors contributing to dysbiosis

**Fig. 1:**
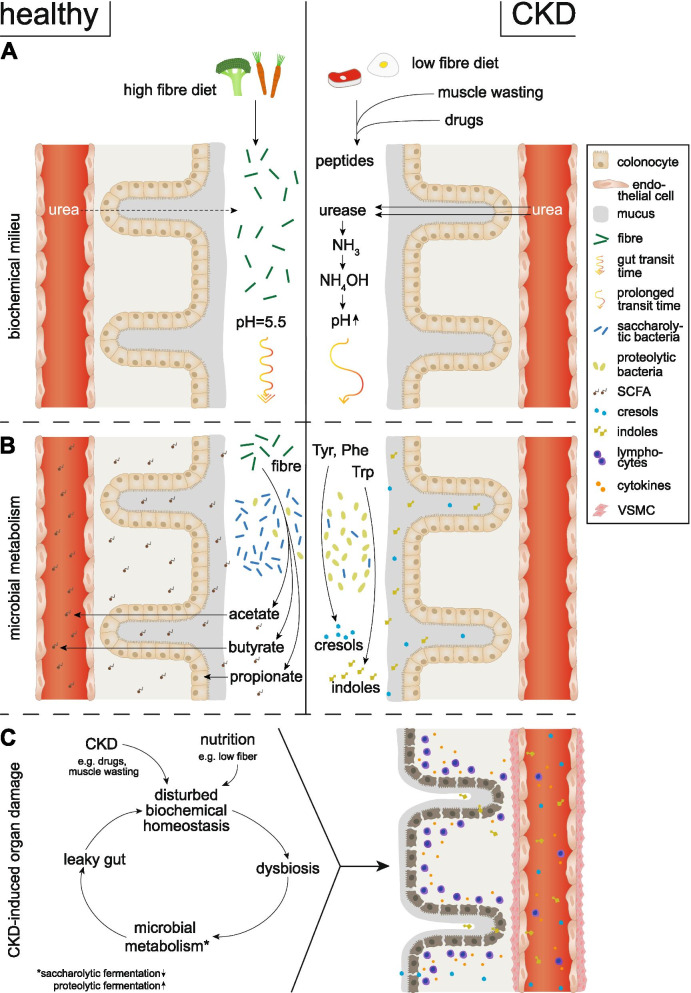
Schematic overview of CKD-specific conditions that influence the local biochemical milieu in the colon (**A**) and contribute to alterations in the composition and metabolism of the gut microbiome (**B**) leading to disruption of the epithelial barrier function (leaky gut), inflammation, and cardiovascular end-organ damage (**C**). **A** In healthy individuals, the biochemical balance in the colon is maintained through a diet rich in fiber providing an energy source for colonocytes and stabilizing local pH as well as downstream metabolism of intestinal microbiota. In contrast, in CKD, several circumstances contribute to a disturbance of biochemical hemostasis. Dietary restrictions (low-fiber diet), oral intake of medications (e.g., antibiotics and phosphate binders), and muscle wasting promote an accumulation of peptides in the gut. Additionally, urea is converted by bacterial-derived urease to ammonium hydroxide which increases the gut pH. Lastly, constipation promotes a prolonged transit time of these metabolites exerting selection pressure on intestinal bacteria. **B** Under normal circumstances, humans benefit from the symbiotic relationship with their gut microbiota by saccharolytic fermentation of complex carbohydrates resulting in the production of SCFA namely acetate, propionate, and butyrate. Whereas butyrate is mainly used by epithelial cells as a nutrient source, acetate and propionate enter the systemic circulation. Generally, SCFAs promote gut epithelial integrity and balance systemic regulation of adaptive immunity and modulate inflammation response. In CKD, dysbiotic changes are characterized by a shift in the fermentation pattern of bacteria from a health-promoting saccharolytic to a proteolytic fermentation. This alteration not only leads to a decrease in local and systemic SCFA concentration but also promotes fermentation of amino acid tryptophan and tyrosine to uremic acid precursors indoles and cresols respectively. **C** Intestinal barrier dysfunction (leaky gut), enforced by dysbiosis and local metabolite imbalance, promotes paracellular migration of bacteria and uremic toxins. Renal excretion decline inevitably induces accumulation of these bacterial-derived metabolites. Moreover, lymphocytes infiltrate the colonocytes’ lamina propria and enter systemic circulation resulting in a state of chronic low-grade inflammation. All these factors finally promote endothelial calcification and cardiovascular disease. CKD chronic kidney disease, Tyr tyrosine, Trp tryptophane, Phe phenylalanine, SCFA short-chain fatty acid, VSMC vascular smooth muscle cell

There are numerous factors contributing to intestinal dysbiosis, which are also summarized in Fig. 1 [80, 117]. Diet is perhaps the most significant environmental factor affecting the gut microbiota in both diseased and healthy individuals. In CKD, dietary restrictions are invariably imposed to prevent fluid overload, hyperkaliemia, and oxalate overload. Most significant with regard to gut health and the microbiome is the low consumption of fruit and vegetables and dietary fiber among CKD patients. This restricted intake of complex carbohydrates reduces the abundance of bacterial phyla capable of utilizing these substrates. Consequently, the normal symbiotic relationship is disturbed, markedly affecting the composition, function, and metabolism of gut microbiota and conferring the potential to significantly alter the biochemical milieu in the CKD population [31, 69, 108].

**Table 1:** Pro- and anti-inflammatory effect of SCFA depending on the SCFA molecule, concentration, and activated pathways

Molecule	Concentration	Pathway	Mediators	Effectors	Reference
**A) Pro-inflammatory effects**					
Butyrate (C4) Propionate (C3)	C4, 2.5–5 mmol/l C3: not specified	GPR43 (FFAR2)	Protein kinase p38α, MAPK phosphorylation, TACE (ADAM17)	ICAM-1↑, E-selectin↑, CINC-2αβ↑ (cytokine-induced neutrophil chemoattractant-2αβ)↑, L-selectin shedding↑	[102, 207]
SCFA unspecified	Not specified	HDAC inhibition	STAT3, NF-κB, and RUNX1	IL-6↑, CXCL1 ↑, and CXCL2 ↑	[99, 208]
Acetate (C2) Propionate (C3) Butyrate (C4)	Not specified	C3/C4: GPR41 (FFAR3); C2/C3: GPR43 (FFAR2)	GPR41 (Gq) -> PLC/Ca^+^ ↑ or PI3K ↑ -> rac/ras -> ERK1/2 or JNK1/3 or p38αMAPK GPR43 (Gi), adenyl cyclase↓, cAMP ↓ -> Ras/Rac ↑ -> ERK1/2 or JNK1/3 or p38α MAPK pathways↑; rapamycin (mTOR) activation of PI3K	IL-6 ↑, CXCL1↑, and CXCL2↑, and interferon gamma (INFγ)↑	[99, 105, 209, 210]
**B) Anti-inflammatory effects**					
Butyrate (C4) > propionate (C3) > acetate (C2)	C4, 46μmol/l C3, 120μmol/l C2, 1–2.4mmol/l	HDAC 1-3 inhibition	MKP-1 acetylation -> dephosphorylation of MAPK, ERK, JNK and p38 MAPK => LPS-induced phosphorylation of p38↓, NF-κB activity↓, eicosanoid production (LOX activation), Mi-2β↑(transcriptional repressor recruited to the IL-6 and TNF promoter); sodium-coupled monocarboxylate transporter 1 (SMCT-1 ) - direct interaction with NF-kB corepressor p65 and p50, deacetylation of p65, enhanced binding of inhibiting molecule IκBα to NF-κB	LPS-induced TNF-α secretion↓ and iNOS expression↓ IL-1β↓, NF-κB activity↓ IL-12 ↓ IL-8↓ CCL2↓, VCAM1↓, MMP2↓, oxLDL uptake↓ CD36↓	[105, 155, 158–161, 170, 172, 176, 211–214]
SCFA unspecified (C3>C4>others)	0.03–5mM	Aryl hydrocarbon receptor (AhR), GPR41 (FFAR3)	Histone deacetylase (HDAC 7, 9, 11) inhibitory activity of the of the Foxp3 promoter; ERK, JNK, and p38 MAPK activation; CTLA-4	adenyl cyclase↓, cAMP ↓; LPS-induced TNFα↓, IL-6↓ and iNOS expression↓; Foxp3↑, Regulatory B cell upregulation => secretion of interleukin-10 (IL-10)↑, IL-35↑, IL-4 ↑ and transforming growth factor-beta (TGF-β)↑, IL 12↓	[103, 104, 171, 172, 215–223]
Acetate (C2) Proprionate (C3) Butyrate (C4)	10mM	GPR43 (FFAR2)	BAX–BAK1–BCL2L1 cluster and the PRKCA–PTPN6–LCK clusters	Induce apoptosis of neutrophils by caspase-8 and caspase-9 pathways	[224, 225]
SCFA unspecified (C2, C3>others)	Not specified	GPR43 (FFAR2) (Gi) heterodimerization and internalization of C5aR and CXCR2, ChemR23	C5a or fMLP (N-formyl peptide f-Met-Leu-Phe); beta-arrestin-2 -> inhibit proteasomal degradation via of IκBα via β-arrestin	Inhibit human neutrophil migration toward C5a or fMLP; cAMP ↓, PLC↑, Ca2+↑	[155, 225]
Butyrate (C4) Propionate (C3)	C4, >0.4 mmol/l C3, >4 mmol/l	HDAC activity, GPR43(FFAR2)	Ca2^+^ mobilization↑, NF-κB↓	TNF-α↓ and CINC-2αβ↓	[155]
Butyrate (C4)	C4, 0.5–10 mmol/l		Nuclear translocation of the p65 subunit↓ NF-κB b↓, peroxisome proliferator-activated receptor–PPAR expression	TNF-α–induced expression of VCAM-1 and ICAM-1	[176, 226]
Propionate (C3) Butyrate (C4)	C3, 12 mmol/l C4, 1.6 mmol/l	GPR109A (Gi)	cAMP↑, NADPH oxidase ↓, reduced phosphorylation of the regulatory subunit NADPH oxidase subunits (p47phox and p22phox); MPO↓	ROS↓ NO↓ superoxide ↓ hydrogen peroxide ↓ and hypochlorous↓	[207, 225, 227]
Acetate (C2) > propionate (C3) Butyrate (C4)	Not specified	GPCR	NALP3 inflammasome activation↑, AMPK activation, MUC2 mRNA expression↑	L-18 production↑, K^+^ efflux↓, Ca2^+^ influx↑, MUC2 secretion, assembly of tight junction proteins ZO-1 and occludin	[228, 229]
SCFA unspecified (C4>others)	Not specified	GPR109A (Gi)	β arrestin-2 -> inhibits proteasomal degradation of IκBα -> NFIB↓, HDAC5, TLR4-; cAMP ↓, PLC↑, Ca2+ ↑	TNF-α↓, IL-6↓, and MCP-1↓	[177, 178, 230–232]

*GPR* G protein-coupled receptor, *FFAR* free fatty acid receptor, *HDAC* histone deacetylase, *ADAM* a disintegrin and metalloprotease, *TACE* tumor necrosis factor-α-converting enzyme, *PLC* phospholipase C, *LPS* lipopolysaccharides, T*NFa* tumor necrosis factor a, *cAMP* cyclic adenosine monophosphate, *PKA* protein kinase A, *PI3K* phosphoinostide 3-kinase, *MAPK* mitogen-activated protein kinase, *MEK* mitogen*-*activated protein kinase kinase, *aMKP* acetylated mitogen-activated protein kinase phosphatase, *ERK* extracellular signal-regulated kinases, *JNK* jun N-terminal kinase, *NF-κB* nuclear factor kappa-light-chain-enhancer of activated B cells, *I-κB* inhibitor of nuclear factor kappa B, *LOX* lipoxygenase, *MCP-1* monocyte chemoattractant protein-1, *ICAM i*ntercellular *a*dhesion *m*olecule*, VCAM* vascular *a*dhesion *m*olecule, *iNOS* inducible nitric oxide synthase, *fMLP* N-*f*ormylmethionyl-leucyl-phenylalanine, *CXCL* chemokine ligand, *AMPK* AMP-activated protein kinase, *NALP* nucleotide-binding oligomerization domain, leucine*-*rich repeat and Pyrin domain containing*, MMP* matrix metalloproteinase, *TLR* toll-like receptor, *ZO* zonula occludens, *MUC* mucin

Moreover, constipation is highly prevalent in CKD. It has been shown that in states where dietary fiber intake is low, as it holds true in CKD, the expansion of proteolytic bacteria may lead to a degradation of the goblet cell-derived mucin 2 (MUC-2) layer, which under normal circumstances facilitates the *nutritional transit by lubricating the gut’s surface.* A slower transit time affects uremic toxin generation by increasing the availability of amino acids to be fermented by proteolytic bacteria [118, 119]. The accumulation of these toxins in turn exacerbates intestinal dysmotility via intestinal inflammation, thereby forming a vicious cycle of constipation coupled with enhanced uremic toxin production, which again increases transit time [97, 120, 121].

In addition, muscle wasting is common in CKD, further exacerbating the advancing issue of uremia, which ultimately leads to increases in colonic pH and dysbiotic alterations. Generally, protein catabolism creates problems in terms of nitrogen elimination and requires conversion to urea within the ornithine cycle in the liver in order to reduce toxicity. The intestinal microbiota can further utilize urea to produce the sulfate-containing amino acids lysine and threonine and thus play a key role in nitrogen recycling [89]. However, in CKD, the rise in retention solute concentrations in the body fluids leads to its massive influx into the gut lumen. Within the intestinal tract, urea is hydrolyzed by microbial urease leading to the formation of ammonia [CO(NH_2_)_2_ + H_2_O → CO_2_ + 2NH_3_]. Ammonia is, in turn, converted to ammonium hydroxide [NH_3_+ H_2_O → NH_4_OH] which elevates the gut’s luminal pH and causes mucosal damage and enterocolitis [109, 122]. Accordingly, it has been proposed that the accumulation of uremic toxins profoundly changes the biochemical environment in the gut and drives a selection pressure that favors microbes capable of using these uremic toxins as substrates by proteolytic instead of saccharolytic fermentation [108–110]. Indeed, data in adults revealed a relative increase in microbes with urease, uricase, and indole- and p-cresyl-forming enzymes [108, 113, 114]. Interestingly, it has been demonstrated that once returned to the typical pH of 5.5 from abnormal high pH levels (6.8), the production of gut-derived uremic acids could be reduced by approximately 33%. Remarkably, this effect was doubled (60% reduction) in the presence of fermentable carbohydrates [123–125].

In addition, pharmaceuticals are known to have an important impact on dysbiosis in CKD. Antibiotics are frequently prescribed in CKD to treat inter alia vascular access infection and account for a considerable loss in key bacteria taxa [61, 62, 126].

Finally, iron-containing phosphate binders to prevent secondary hyperparathyroidism are associated with increased production of uremic toxins and cIMT, which may be due to an iron-dependent expansion of the phylum Proteobacteria and a general decrease in the abundance of beneficial saccharolytic species such as *Lactobacillus* and *Bifidobacterium* [108, 126–128].

## Crosstalk between the gut microbiome and target organs in CKD via microbially derived metabolites

### Microbially derived uremic toxins as drivers of cardiovascular disease

In CKD, the uremic syndrome is attributed to the progressive loss of excretory function, which inevitably results in the retention of a variety of substances. These retention solutes are found to exert toxicity that affects numerous biological functions, hence referred to as uremic toxins [129]. According to the European Uremic Toxin (EUTox) Work Group, they can be classified into three physicochemical categories based on the molecular weight and kinetic behavior during dialysis. Besides small water-soluble molecules (molecular weight (*MW*) ≤ 500 Da—i.e., urea and phosphorus) and middle-sized molecules (*MW* ≥ 500 Da—i.e., parathyroid hormone and β_2_-microglobulin), the third group consists of plasma-bound compounds like indoxyl sulfate (IS) and p-cresol sulfate (PCS). The latter have long been neglected with respect to their pathophysiological importance despite their tendency to accumulate during dialysis due to being mostly albumin bound and therefore not filtered out [27, 130, 131].

Uremic toxins mainly result from proteolytic fermentation of amino acids in the intestine. Therefore, the generation of these toxins depends mainly on nutritional intake and on microbial dysbiosis in CKD, favoring proteolytic fermentation as described above. The role of the microbiota in the generation of uremic toxins has been emphasized in a study of hemodialysis patients who received a colectomy. The authors demonstrated the absence of more than 30 uremic toxins in this study group highlighting the colon microbes as a prime contributor to the uremic milieu present in CKD patients [132].

Among the metabolic pathways associated with uremic toxicity, tryptophan (TRP) metabolism has been the focus of numerous studies (Fig. 1). Although tryptophan is an essential amino acid, less than 1% is used for protein synthesis. The vast majority serves as a biosynthetic precursor for distinct microbial metabolization pathways generating serotonin, melatonin, kynurenine, and indoles [133]. Approximately 95% of TRP is converted to kynurenine (KYN) by the enzymes tryptophan 2,3-dioxygenase (TDO) and indoleamine 2,3-dioxygenase (IDO). In adult CKD, metabolites of the KYN pathway such as kynurenic acid and xanthurenic acid have been linked to chronic inflammation [134, 135], mineral bone disease [136], thrombosis [137, 138], and cognitive impairment [135]. A variable portion of dietary TRP is catabolized to indole metabolites by intestinal bacteria, which express enzymes such as tryptophanase [139]. Indoles are further metabolized by gut microbiota and the liver to indoxyl-sulfate (IS), the prototype of microbially derived uremic toxins [140].

The accumulation of uremic toxins in adult CKD patients has been repeatedly described and is associated with the progression of kidney disease and its comorbidities, first and foremost CVD [141–143]. Subsequently, pediatric nephrologists started to pay attention to these gut-derived uremic toxins and their impact on CKD-associated comorbidities. Initial studies showed that hippuric acid (HA), indole acetic acid (IAA), IS, and PCS were indeed elevated in pediatric CKD patients [131, 144]. Recently, further investigations provided insights into the clinical implications of elevated serum levels of uremic toxins in pediatric CKD. Our work has demonstrated that IS concentration not only inversely correlates with the estimated glomerular filtration rate (eGFR) but also associates with disease progression and cardiovascular morbidity, independent of other known risk factors like proteinuria or blood pressure [127, 145].

While not yet shown in children, detailed mechanisms of the accumulation of uremic toxins leading to CVD in adult CKD patients have been demonstrated [27, 141, 146–149]. In brief, the uremic milieu provokes endothelial production of reactive oxygen species (ROS) leading to a pro-inflammatory state in which the NOD-like receptor proteins (NLRP3) of the inflammasome are activated and downstream cytokine production promoted (e.g., IL-6). The downstream transcription of several adhesion molecules ultimately results in endothelial dysfunction [150]. IS is perhaps the best characteristic of the gut-derived uremic toxins and recent work focusing on this metabolite suggests its interfering role regarding oxidative stress levels as it was shown to stimulate the generation of free radicals by activation of NADPH oxidase [151]. Moreover, IS induces vascular smooth muscle cell (VSMC) proliferation, reduces endothelial repair, and promotes vascular calcification via its binding to the nuclear aryl hydrocarbon receptor (AhR) and activation of the NF-κB signaling pathway [152–154]. In the clinical context, studies point toward an association of serum IS levels with surrogate markers of CVD, namely cIMT and PWV [127].

### Decrease of SCFA induces a pro-inflammatory and proatherogenic milieu in CKD

The reduced abundance of SCFA-producing bacteria in the gut of CKD patients is coupled with a marked decrease in SCFA in the blood (Fig. 1) [31]. SCFAs are key regulators of inflammation via a number of interactions. They can modify the transcription of genes responsible for leukocyte rolling and subsequent adhesion and migration of immune cells. Additionally, they play an essential role in controlling the production of pro-inflammatory cytokines. The binding of SCFA to different intracellular and extracellular receptors is discussed below, and potential mechanistic insights into the link between gut-derived metabolites and their potential to control immune function are highlighted [105].

SCFAs, particularly butyrate and propionate, are shown to be effective non-competitive inhibitors of the histone deacetylase (HDAC) enzyme in a millimolar range, which lays within the physiologic concentration of SCFA in the lumen of the colon (from 20–70 mmol/l proximally to 70–140 mmol/l distally) [94, 155, 156]. Interestingly, evidence shows that these two SCFAs can modulate endothelial and immune cell inflammation by inhibition of HDAC [99, 105, 157, 158]. Recent studies revealed the potential of SCFAs to lower levels of LPS-induced TNFα production in mononuclear cells via inhibition of NF-κB [159]. In detail, deacetylation of p65, a subunit of NF-κB, enhances its binding to the inhibiting molecule IκBα in the nucleus and may result in the export of NF-κB complexes back to the cytoplasm, where it cannot deploy its transcriptional pro-inflammatory activity [160]. Importantly, the order of potency for NF-κB suppression butyrate > propionate > acetate coincides with the order of HDAC suppression activity [161, 162].

It has been observed that HDAC inhibitors induce IL-10 gene expression in regulatory T cells (Tregs) and elevate suppression capacity of Tregs thereby providing further mechanistic insights explaining SCFA’s anti-inflammatory capacity, as colonic Tregs limit proliferation of effector CD4+ T cells (Teff) and thus control inflammation [163]. More specifically, Clostridia species known as high butyrate producers are the most potent inducers of de novo generation of inducible colonic Tregs (iTregs) in the large intestine [164–166]. In germ-free mice, SCFAs, in particular propionate (C3), increase both the absolute count and the proportion of Tregs and augment their immune suppressive function [104]. Current data reveal the potential of SCFA to induce the production of anti-inflammatory cytokines such as IL-10 and the repression of pro-inflammatory molecules IL-12, TNFα, IL-1β, and NO by inhibiting NF-κB activity [101, 103, 155, 159]. Moreover, the most potent inhibitor of HDACs butyrate (80% inhibitory efficiency) protects against vascular inflammation and atherosclerosis, thereby modulating oxidative stress and endothelial function [167, 168]. Still, the broad spectrum of HDACs with pleiotropic and even divergent effects on transcriptional responses should be kept in mind, bearing future topics of research, e.g., if and by which pathways different types of HDAC mediate the effects of SCFAs on colonocytes and immune cells.

SCFA may also act through binding to specific membrane-bound receptors. In this context, two free fatty acid receptors (FFA-R) are discussed in more detail. In both cases, the carbon molecule chain length dictates their respective activation potential. SCFAs acetate and propionate bind to FFA2-R (G protein-coupled receptor, GPR43) that is primarily expressed on neutrophils, eosinophils, dendritic cells, and monocytes suggesting their role in inflammatory responses [169]. Activation of FFA2-R results in an attenuated NF-κB response with downregulated release of pro-inflammatory cytokines, including IL-6 and IL-1beta. This effect may be mediated by β-arrestin—a regulator of G protein-coupled receptors, as demonstrated by restored cytokine production upon β-arrestin-2 knockout [170]. Conversely, FFA3 receptors (GPR41) are most efficiently activated by C3-C4 SCFAs; thus, propionate and butyrate present the main activators [171, 172]. FFA3 receptors are infrequently expressed on immune cells but rather on the surface of the pancreas, spleen, and adipose tissue [172]. Their implications in obesity and metabolic disorder are widely recognized [172]. Despite the lower abundance of FFA3 receptors on immune cells, studies still suggest an anti-inflammatory potential of butyrate and propionate. In short, activation of the FFA3 receptor decreased LPS-induced TNFα expression as well as MCP-1, IL-6, and inducible nitric oxide synthase (iNOS) levels naturally released by monocytes and neutrophils [105, 155, 173].

At the same time, data from recent studies emerged pointing toward a pro-inflammatory effect of FFA2-R and FFA3-R activation accompanied by increased production of IL-6, CXCL1, and CXCL2 [99].

Data on SCFAs and their influence on immune cell migration are sometimes contradictory. While some research shows induction of neutrophil recruitment by SCFAs followed by migration to the cite of inflammation via binding to FFA2-R following MAPK activation, other studies demonstrate the capacity of butyrate to suppress the expression of adhesion molecules such as VCAM-1, ICAM-1, lymphocyte function-associated antigen-3 (LFA-3), and L-selectin [100, 102, 155, 174, 175] via inhibition of histone acetylation and NF-κB-suppression. As this may lead to a lower incidence of adhesion and migration of macrophages to vascular lesion areas, it is tempting to speculate the potential of SCFAs as an influential mediator in the prevention of arteriosclerosis [176].

Concerning vascular inflammation, another GPR109A also known as hydroxyl-carboxylic acid 2 (HCA2) has been reported to decrease cytokine secretion of TNFα, IL-6, and MCP-1 and, as a result, alleviate the effect on the progression of arteriosclerosis. Of the main SCFAs, butyrate shows the highest potential for GPR109A activation and is shown to inhibit ROS production [105, 177, 178].

**Fig. 2:**
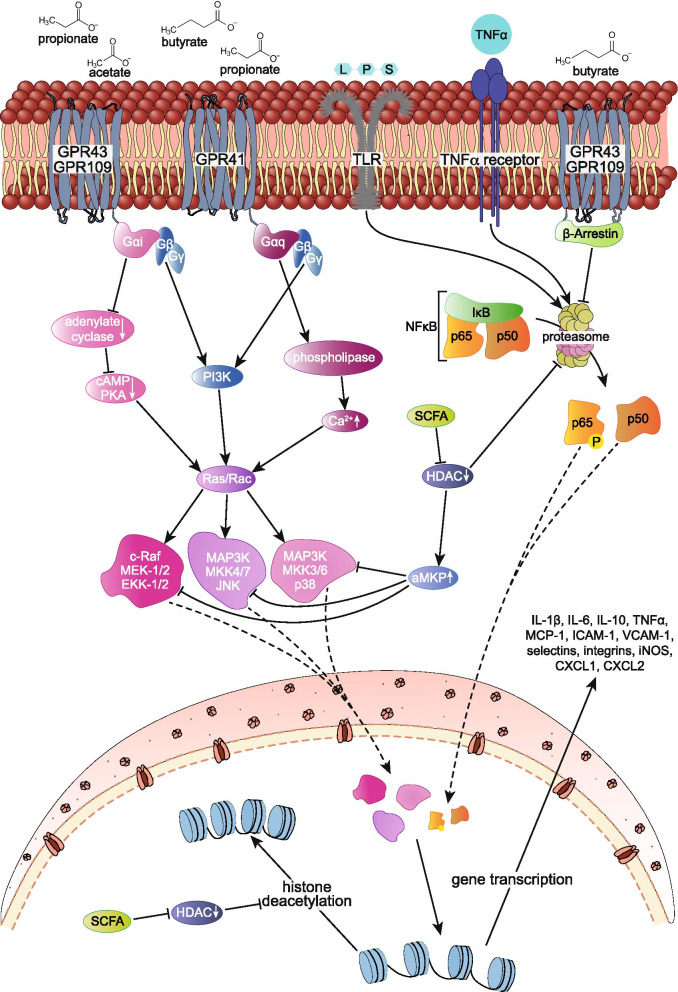
Overview on the effect of SCFAs, LPS, and TNFα on gene transcription of inflammatory cytokines and adhesion factors involved in immune cell activation and promotion of endothelial dysfunction. LPS and TNFα bind to their receptor and activate downstream MAPK and NF-κB signaling through phosphorylation and subsequent ubiquitination of I-κB (inhibitory subunit of NF-κB) resulting in its degradation thus inducing gene transcription of pro-inflammatory molecules. SCFA pleotropic effects on inflammation are mediated through binding of extracellular G protein-coupled receptors as well as its potential to inhibit histone deacetylation. Acetate and propionate primarily bind to FFA2-R/GPR43 which are expressed on immune cells and may inhibit proteasomal degradation of I-κB via β-arrestin attenuating NF-κB response. Additionally, contrary effects of FFA2-R/GPR43 activation are shown as SCFA binding initiates downstream MAPK signaling. SCFA butyrate dictates activation of G protein-coupled receptor FFA3-R/GPR41 and GPR109 which decreases LPS-induced expression of TNFα, MCP-1, IL-6, and inducible nitric oxide synthase (iNOS). Confliction to its anti-inflammatory effect butyrate is also shown to enhance MAPK signaling via its binding to extracellular G protein-coupled receptor FFA3-R/GPR41. Despite receptor signaling, SCFAs are potential non-competitive inhibitors of histone deacetylases (HDACs). These enzymes facilitate a decrease in the interaction of histones to the DNA, hence promoting gene transcription. Furthermore, HDAC enhances acetylation status and thus inhibitory interaction potential of MKP-1 to MAPK and in this way attenuates downstream pro-inflammatory gene transcription. Lastly, SCFAs may decrease proteasome activity through HDAC and inhibit TNFα-induced NF-κB activation normally mediated by the degradation of I-κB. GPR G protein-coupled receptor, LPS lipopolysaccharides, TNFa tumor necrosis factor a, cAMP cyclic adenosine monophosphate, PKA protein kinase A, PI3K phosphoinostide 3-kinase, MAPK mitogen-activated protein kinase, MEK mitogen-activated protein kinase kinase, aMKP acetylated mitogen-activated protein kinase phosphatase, HDAC histone deacetylase, NF-κB nuclear factor kappa-light-chain-enhancer of activated B cells, I-κB inhibitor of nuclear factor kappa B, MCP-1 monocyte chemoattractant protein-1, ICAM intercellular adhesion molecule, VCAM vascular adhesion molecule, iNOS inducible nitric oxide synthase, CXCL chemokine ligand

To conclude, inflammatory regulation properties of SCFA on endothelial and inflammatory cells within the human gut are complex. A growing body of evidence suggest their potential to modulate inflammation, cytokine production, and immune cell migration likely mediated through binding of extracellular G protein-coupled receptors and inhibition of histone deacetylases which regulate gene transcription, which is summarized in Fig. 2. However, due to the pleiotropic effects of SCFA, which are summarized in Table 1, the exact mechanisms by which they promote or inhibit inflammation and consequently CVD remain elusive.

### Vicious cycle of uremia, loss of gut symbiosis, and disrupted barrier functions (leaky gut)

The intestinal epithelium is at the center of interactions between the immune system and luminal content of the gut including microbes, microbial products, and dietary compounds. Hence, its primary role is to maintain balance whereby invasion of pathogens is prevented and simultaneously provide selective permeability allowing immune stimulation and nutritional uptake. Disruption of epithelial integrity leads to local and systemic inflammation and is intensively discussed to modulate the initiation and progression of several diseases [179].

As paracellular transport is generally more permeable than a transcellular pathway, structural components, particularly the tight junction proteins occludin and claudin, are principal determinants of gut integrity and prevent unregulated passing of nutritional antigens and microbes [31, 180, 181]. In uremic CKD patients, barrier dysfunction is common and there is mounting evidence for its pivotal role in the pathogenesis of systemic low-grade inflammation as well as its contribution to end-organ damage. Leaky gut facilitates endotoxemia with no clinical signs of infection and massive infiltration of mononuclear leukocytes in the lamina propria accompanied by a marked thickening of the colonic wall [182]. Further evidence for the leaky gut phenotype in CKD includes the detection of bacteria DNA fragments in the intestinal wall [26, 183, 184], reduced transepithelial electrical resistance in vitro following incubation with a pre-dialysis plasma of ESKD patients which can be rescued with post-dialysis plasma [185], and depletion of transcellular and intracellular protein constituents of the tight junction [31].

Several mechanisms are likely to mediate the progressive breakdown of the apical junctional complex. Firstly, as SCFA production declines, the colonic epithelia are effectively starved of the essential nutrition that jeopardizes the health of the mucosal cells. Secondly, alterations in the biochemical milieu are associated with an accumulation of urea-derived NH_3_ and ammonium hydroxide. In fact, twelve of the 19 microbial families (63%) with the greater abundances in ESKD patients were among the urease-possessing families. Recent studies demonstrated their potential to be a caustic compound capable of dissolving proteins, which subsequently weakens their ability to seal the intercellular space. Hence, it allows a now unrestricted transport of large solutes like dietary antigens and bacterial lipopolysaccharides thus aggravating systemic inflammation [185–187].

Furthermore, fluid overload, generalized edema, and congestive heart failure are common complications in CKD all of which can aggravate endotoxemia and contribute to a disturbance in mucosal barrier function [188, 189]. In addition, aggressive ultrafiltration or generous use of diuretics can lead to hypotension and bowel ischemia impacting the intestinal epithelia [190]. As the microbial flora is highly sensitive to any changes in the iron concentration, gastrointestinal micro-bleeding followed by uremic platelet dysfunction and high incidence of angiodysplasia and systemic anticoagulation disrupt the barrier function [191].

The molecular events that cause increased permeability are poorly defined. Recent work suggests that myosin light chain kinase (MLCK) plays a central role in epithelial barrier hemostasis, which is disrupted by chronic inflammation both in vivo and in vitro [192].

Of note, inflammation and disruption of the epithelial tight junction in CKD is associated with an impaired anti-oxidative system demonstrated by a decrease in the key antioxidant enzymes catalase and Cu-Zn superoxide dismutase on the one hand, and increased plasma level of nitric oxide synthase, monocyte chemotactic protein 1, and COX-2 on the other. These oxidative stress mediators induce the depletion of epithelial tight junction proteins, i.e., ZO-1, occludin, and claudin-1 [193].

In summary, leaky gut is an important hallmark in CKD pathophysiology, causally linked to dysbiosis and a loss of local metabolite hemostasis, being itself aggravating the detrimental interaction between the gut microbiota and the host by means of a vicious cycle (Fig. 1).

## Conclusions and outlook: targeting the gut microbiome to attenuate CVD and progression of CKD in children

A novel additional approach in the treatment strategy of CKD could target gut health rather than the primary diseased organ [194]. In light of all the mechanistic evidence described here, a logical next phase in improving health outcomes for CKD patients would be to treat the gut microbiome dysbiosis, reduce bacterial production of uremic toxins, and boost SCFA production. Potential exists for dietary strategies to mitigate the downstream consequences of microbiome dysbiosis that drive disease progression in CKD, such as the gut barrier dysfunction, systemic low-grade inflammation, and accumulating uremic toxins [77, 195, 196].

A large-scale epidemiological study with CKD patients has recently identified a diet low in fruits and vegetables as a significant risk factor for ESKD [197]. Fiber shortens intestinal transit time and promotes the growth of saccharolytic bacteria. The particular benefit of this for CKD patients is by reducing the time for absorption of the metabolites of proteolytic fermentation and most significantly by increasing SCFAs to support a healthy intact epithelial barrier. Fiber deficiency is common in CKD patients, who tend to avoid fruits and vegetable in a misguided attempt to prevent diet-induced hyperkalemia that can have severe consequences for patients with impaired renal function [198].

Therefore, in the simplest terms, more fruits, vegetables, and dietary fiber should be included in the typical CKD diet, but caution should be implemented due to the risk of hyperkalemia. Individualized dietary plans are required with restriction to low potassium vegetables and fruits when appropriate.

Furthermore, pre- and probiotic supplementation could potentially help to improve gut health in CKD as well as other chronic diseases. Prebiotics have been defined as “substrates that are selectively utilized by host microorganisms conferring a health benefit” [199]. Indeed, a small trial in hemodialytic adults showed a significant reduction in circulating indoxyl sulfate and *p*-cresyl sulfate levels following oligofructose-inulin or resistant starch supplementation [200, 201]. However, attempts to restore the desired saccharolytic bacteria by introducing favorable microorganisms (probiotics) failed to reduce plasma concentrations of uremic solutes. The lack of benefit with probiotics may be partially explained by a persistence of uremia-induced biochemical milieu; thus, attempts with probiotic formulations without simultaneously improving the biochemical environment in the gut will be of no avail [31, 202]. The use of pre- and probiotics in patients with CKD has been reviewed recently confirming these contradictory findings [203].

Another promising approach is the use of oral adsorbents. AST-120, a highly potent charcoal that is widely known as a decontaminant, has been shown to markedly reduce the plasma concentration of indoxyl sulfate and p-cresol sulfate [204]. In animals, the adsorbent partially restored expression of tight junction proteins in the colon, reduced monocyte activation, and lowered inflammatory markers such as endotoxin, IL-6, and TNF-α [187]. However, AST-120 had no significant effect in terms of slowing CKD progression [205, 206].

To the best of our knowledge, no large-scale interventional studies have investigated the use of pre- or probiotic supplementation in children with CKD. Nevertheless, a small observational study in children with CKD revealed an inverse association between fiber consumption and serum concentrations of several protein-bound uremic toxins such as indoxyl sulfate, p-cresyl sulfate, indole acetic acid, and p-cresyl glucuronide [202]. In the absence of other contributing diseases which are commonly seen in adults, pre- or probiotic supplementation and improved, individualized nutrition seem to be a very promising treatment to slow CKD progression and prevent CKD-associated comorbidities.

## Data Availability

Not applicable.
